# Liver Trauma: New Management Trends

**DOI:** 10.1155/1994/19580

**Published:** 1994

**Authors:** J. M. Little

**Affiliations:** Department of Surgery Westmead Hospital Westmead N.S.W. 2145 Australia


*HPB Surgery*, 1994, Vol. 7, pp. 251–259
Reprints available directly from the publisher
Photocopying permitted by license only
© 1994 Harwood Academic Publishers GmbH
Printed in the United States of America

### HPB INTERNATIONAL

**EDITORIAL & ABSTRACTING SERVICE**

JOHN TERBLANCHE, EDITOR
Department of Surgery,
Medical School · Observatory 7925
Cape Town · South Africa
Telephone (021) 406-6232 Ext. 2
Telefax (021) 448-6461

### LIVER TRAUMA: NEW MANAGEMENT TRENDS

### ABSTRACT

*Pachter, H.L., Spencer, F.C., Hofstetter, S.R., Liang, H.G., Coppa, G.F. (1992) Significant trends in the treatment of hepatic trauma. Annals of Surgery; 215: 492–502.*

**Several significant advances in the treatment of hepatic injuries have evolved over the past decade. These trends have been incorporated into the overall treatment strategy of hepatic injuries and are reflected in experiences with 411 consecutive patients. Two hundred and fifty-eight patients (63%) with minor injuries (grades I to II) were treated by simple suture or hemostatic agents with a mortality rate of 6%. One hundred and twenty-eight patients (31%) sustained complex hepatic injuries (grades III to V). One hundred seven patients (83.5%) with grades III or IV injury underwent portal triad occlusion and finger fracture of hepatic parenchyma alone. Seventy-three surviving patients (73%) required portal triad occlusion, with ischemia times varying from 10 to 75 minutes (mean, 30 minutes). The mortality rate in this group was 6.5% (seven patients) and was accompanied by a morbidity rate of 15%. Fourteen patients (11%) with grade V injury (retro-hepatic cava or hepatic veins) were managed by prolonged portal triad occlusion (mean cross-clamp time, 46 minutes) and extensive finger fracture to the site of injury. In four of these patients an atrial caval shunt was additionally used. Two of these patients survived, whereas six of the 10 patients managed without a shunt survived, for an overall mortality rate of 43%. Over the past 4 years, six patients (4.7%) with ongoing coagulopathies were managed by packing and planned re-exploration, with four patients (67%) surviving and one (25%) developing an intra-abdominal abscess. One additional patient (0.8%) was managed by resectional debridement alone and survived. During the past 5 years, 25 hemodynamically stable and alert adult patients (6%) sustaining blunt trauma were evaluated by computed tomography scan and found to have grade I to III injuries. All were managed nonoperatively with uniform success. The combination of portal triad occlusion (up to 75 minutes), finger fracture technique,**

**and the use of a viable omental pack is a safe, reliable, and effective method of managing complex hepatic injuries (grade III to IV). Juxtahepatic venous injuries continue to carry a prohibitive mortality rate, but nonshunting approaches seem to result in the ?? cumulative mortality rate. Packing and planned reexploration has a definitive life-saving role when used adjunctively.**

### PAPER DISCUSSION

KEY WORDS: Liver trauma

The authors are to be congratulated on this excellent review of available methods of management of liver trauma. The article should be obligatory reading for all of those who look after liver trauma.

The authors have analysed their experience with 411 liver injuries. They use a grading system which extends from Grade I for the most minor haematoma or laceration to Grade VI which represents avulsion of the liver from the inferior vena cava. Grade V injuries represent more than 75% destruction of a hepatic lobe or a juxta-hepatic venous injury. 258 of the injuries encountered between 1977 and 1991 by the authors in New York were of Grade I or II severity. These were dealt with by simple methods, such as suture or application of haemostatic agents. The results, as would be expected, were excellent, with a mortality of only 6%. 128 of the injuries were more complex, in Grades III to V. The great majority of the more complex injuries (83%) were graded as either III or IV, and these injuries were usually dealt with by occlusion of the portal triad and finger fracture of liver parenchyma to allow access for haemostasis and drainage. This approach was introduced by the authors[1], and it has clearly served them well, since the mortality in this group was only 6.5%. It appears from this report that portal triad occlusion can be safely maintained, even in the presence of liver trauma, for up to 75 minutes. For those practising outside North America, where blunt injury is likely to be more important than penetrating injury, it is important to recognise that 83% of these Grade III or Grade IV injuries resulted from penetrating injury by stab or gunshot.

Fourteen patients (3.4% of the total series) suffered Grade V injury to the juxtahepatic venous structures. Once again, it is important to note that 9 of these came from penetrating injuries and 5 from blunt tears. To cope with these drastic injuries, the authors employed atriocaval shunts on 4 occasions with 2 survivors, and a direct approach by portal clamping and finger fracture in 9 patients with 6 survivors. One patient with Grade V injury died before surgery.

The authors have also used perihepatic packing in desperate situations, with planned re-operation. The use of this mode of treatment has been limited, however, to only 6 patients in the space of 4 years. There has been little use made of resectional debridement, and only one patient has been treated in this way in the present series. By contrast, patients who were haemodynamically stable with computerised tomographic (CT) evidence of hepatic trauma, have been treated non-operatively, and in this group the authors have accumulated 25 patients in five years without mortality.

This admirable series encapsulates a model North American approach to an important problem. The authors have used a good and intelligible grading system, which makes it relatively easy to compare their results with those of other authors. There still remains, however, a need for an internationally recognised grading system, since each major group dealing with liver injuries seems to come up with something that is different from that of other workers.

The present paper confirms current views on non-operative management[2,3]. This method is applicable to patients with isolated liver injuries or multiple injuries provided that they are haemodynamically stable and provided that there are excellent organ imaging facilities readily available and provided that intensive monitoring can be provided. The trend towards conservative surgery with avoidance of resection wherever possible is also confirmed by this paper. The authors first reported the finger fracture technique[1] which allows the liver to be opened for access to bleeding vessels, avoiding the need to resect liver tissue. The material on portal clamping is also useful in confirming the safety of clamp times beyond 60 minutes, even in severe liver injury[4]. The subject of juxtahepatic venous injury deserves special comment. The authors have achieved excellent results with their technique of portal triad clamping and finger fracture approach to the area of venous damage. The low incidence of juxtahepatic venous injury is surprising. The overall incidence of 14 patients with venous injury in 411 patients with liver injury represents only 3.4%. Schrock and Blaisdell[5] suggested that venous injury occurred in about 15% of patients with liver injuries in North America, while Hollands and Little[6] reported a 13% incidence in 306 predominantly blunt liver injuries. The present authors have reported 8 survivors in their 14 grade V injuries, and this seems to be as good a result as any achieved elsewhere in the world. Ciresi and Lim[7] have continued to advocate atriocaval shunts, and their results are similar to those reported in this present series. It is worth noting, however, that the Ciresi and Lim[7] series is somewhat larger than the present series, and that the majority of survivors experienced penetrating trauma.

Krige, Worthley and Terblanche[8] have reported a simplified approach to total isolation of the liver by caval clamping and portal triad clamping for a prolonged time, and it may well be that this relatively simple technique will provide more rapid control than the insertion of an atriocaval shunt.

Hollands and Little[6] have drawn attention to a distinction which should be made between injuries in which the trunk of a hepatic vein is torn from the inferior vena cava and those which involve a tear of the hepatic vein secondary to avulsion of a major branch. The mortality of venous trunk avulsion seems to be about 75%, compared with about 30% for avulsion of a major branch. It would seem important that authors in future supply anatomical data of this kind.

Liver injury continues to be an important and mortal lesion. The authors of this present article have reviewed the repertoire of management methods, and once again re-emphasised the value of their finger fracture technique. Their paper reflects the general trend towards conservatism and emphasises the central value of organ imaging in diagnosis and management. In addition, it re-emphasises the value of packing as a salvage manoeuvre and endorses the safety of long term occlusion of portal inflow during the management of severe injuries.

### REFERENCES

1. Pachter, H.L., Spencer, F.C. and Hofsetter, S.R. (1983) Experience with the finger fracture technique to achieve intrahepatic hemostasis in 75 patients with severe injuries to the liver. *Ann.Surg.*, **197**, 771–777

2. Delius, R.E., Frankel, F. and Coran, A.G. (1989) A comparison between operative and non-operative management of blunt injuries to the liver and spleen in adult and pediatric patients. *Surgery*, **106**, 788--793
3. Hollands, M.J. and Little, J.M. (1991) Non-operative management of blunt liver injuries. *Brit.J.Surg.*, **78**, 968–972
4. Kahn, D., Hickman, R., Dent, D.M. and Terblanche, J. (1986) For how long can the liver tolerate ischaemia? *Eur.Surg.Res.*, **18**, 277–282
5. Schrock, T. and Blaisdell, F.W. (1968) Management of blunt trauma to the liver and hepatic veins. *Arch.Surg.*, **96**, 698–704
6. Hollands, M.J. and Little, J.M. (1990) Hepatic venous injury after blunt abdominal trauma. *Surgery*, **107**, 149–152
7. Ciresi, K.F. and Lim, R.C. Jr. (1990) Hepatic vein and retrohepatic vena caval injury. *World J.Surg.*, **14**, 472–477
8. Krige, J.E.J., Worthley, C.S. and Terblanche, J. (1990) Severe juxtahepatic venous injury: survival after prolonged hepatic isolation without shunting. *HPB Surgery*, **3**, 39–45

J. M. Little
Department of Surgery
Westmead Hospital
Westmead N.S.W. 2145
Australia

### GROWTH AND SURVIVAL IN SMALL HEPATOCELLULAR CARCINOMA

### ABSTRACT

*Barbara, L., Benzi, G., Gaiani, S., Fusconi, F., Zironi, G., Sirongo, S., Rigamonti, A., Barbara, C., Grigioni, W., Mazziotti, A., Bolondi, L. (1992) Natural history of small untreated hepatocellular carcinoma in cirrhosis: A multivariate analysis of prognostic factors of tumour growth rate and patient survival. Hepatology; 16: 132–137.*

**We analyzed the growth pattern of tumor masses and the survival of 39 asymptomatic Italian patients with a total of 59 small (≤ 5 cm in diameter) hepatocellular carcinomas arising from cirrhosis. The total length of the observation period ranged from 90 to 962 days, with an average of 364 ± 229 (mean ± S.D.). Doubling time ranged from 27.2 to 605.6 days (mean ± S.D., 204.2 ± 135; median = 171.6 days). Three different growth patterns were recognized: (a) tumors with no or very slow initial growth pattern (doubling time > 200 days), 10 cases (37%); (b) tumors with declining growth rate over time, 9 cases (33.4%); and (c) tumors with almost constant growth rate, 8 cases (29.6%). Using the stepwise discriminant analysis, we found a score based on albumin, alcohol intake, number of nodules, echo pattern and histological type that allowed a correct prediction of short doubling time (≤ 150 days) in 55.6%, medium doubling time (151 to 300 days) in 60% and long doubling time (> 300 days) in 100% of cases. The estimated survival rate of the 39 patients,**

